# Transcriptome Sequencing Revealed an Inhibitory Mechanism of Recombinant Puroindoline B Protein on *Aspergillus flavus*

**DOI:** 10.3390/foods14111903

**Published:** 2025-05-27

**Authors:** Pingping Tian, Cuixiang Li, Yangyong Lv, Shaobin Gu, Yuansen Hu

**Affiliations:** 1 College of Food and Bioengineering, Henan University of Science and Technology, Luoyang 471023, China; 2 College of Biological Engineering, Henan University of Technology, Zhengzhou 450001, China

**Keywords:** Puroindoline B protein, *Aspergillus flavus*, RNA-seq, major facilitator superfamily transporters

## Abstract

*Aspergillus flavus*, a common food contaminant, poses health and economic risks. Previous research showed that recombinant Puroindoline B protein (rPINB) inhibited *A. flavus* by disrupting its cell wall, membrane, nuclear function, mitochondrial activity, and oxidative stress. This study used transcriptome technology to explore the impact of rPINB on *A. flavus* gene expression and created gene deletion strains to test the sensitivity to rPINB. RNA-Seq identified the differentially expressed genes (DEGs) affecting cell wall synthesis, membrane transport, oxidative stress, spore formation, and aflatoxin production. The MFS transporter genes AFLA_106900 (*mfs1*) and AFLA_106910 (*mfs2*) were crucial for an inhibitory effect of rPINB. The mutants exhibited reduced sensitivity to rPINB-mediated inhibition, indicating lower growth, sunken conidia, and shriveled hyphae, compared to the wild-type strain. The results also demonstrated decreased sensitivity to the stress agents affecting cell membranes, osmotic balance, and oxidation, alongside a significant reduction in AFB1 production in gene-deleted strains. These results suggested that *mfs1* and *mfs2* were essential for rPINB protein’s inhibition of *A. flavus* growth, laying the groundwork for the mold control strategies using plant proteins.

## 1. Introduction

*Aspergillus flavus*, a common fungal species, has the capability to invade and colonize agricultural commodities at multiple stages, including pre-harvest, harvest, and storage phases [1,2]. This fungus may manifest as either spores or mycelium, residing internally or externally on these agricultural products [3,4]. Under conducive environmental conditions, characterized by optimal temperature, humidity, and pH, *A. flavus* can proliferate rapidly, leading to contamination that diminishes both the economic value and societal utility of the affected products. Furthermore, the secondary metabolites produced by *A. flavus*, particularly aflatoxins, present a substantial threat to the quality, nutritional integrity, and safety of these products for human consumption [5,6]. While chemical inhibitors, such as the silver hydrogen peroxide ion complex disinfection system, and microbial antagonism techniques, like the biological competition between *Bacillus* species and *Lactobacillus*, are continually being refined, it remains essential to develop a comprehensive mildew control network that is aligned with the life cycle of crops [7,8,9,10].

Postharvest wheat grains maintain an active metabolic state, which leads to the production of secondary metabolites such as plant defense hormones and pathogenesis-related (PR) proteins [11,12]. The Puroindoline B (PINB) protein plays a crucial role in regulating grain hardness in wheat, characterized by a molecular weight of approximately 15 kDa and a tryptophan-rich domain (TRD) [13,14]. The TRD domain interacts with the cell membrane through hydrophobic interactions, serving as the primary region responsible for its antibacterial properties [15,16]. Previous research had demonstrated that puroindoline proteins possess the ability to inhibit the growth of molds, including *A. glaucus*, *A. flavus*, and other fungi commonly associated with grain storage [17,18].

Recent advancements have enhanced the understanding of the antifungal mechanisms of the PINB protein. Our research team has confirmed, using propidium iodide staining and scanning electron microscopy, that the recombinant PINB protein (rPINB) disrupts microbial membrane integrity, resulting in the leakage of cellular contents. Additionally, rPINB induced oxidative stress and triggered early mitochondrial apoptosis [19,20]. However, the molecular mechanism of rPINB protein function remains unclear. This study specifically targeted *A. flavus* as the subject of investigation. Transcriptomic analysis was utilized to explore the antifungal mechanism of the rPINB at the gene expression level, with validation achieved through homologous recombination. The findings of this research further elucidate the antifungal properties of wheat kernels and provide a foundational basis for the development of mold control strategies leveraging plant-derived proteins.

## 2. Materials and Methods

### 2.1. Fungal Strains and Growth Conditions

The *A. flavus* strain NRRL3357 was kindly provided by Professor He Zhumei from the School of Life Sciences at Sun Yat-sen University (Guangzhou, China). The spores were cultured on potato dextrose agar (PDA) medium, comprising 20% potato, 2% glucose, and 2% agar, at 30 °C for a duration of five days. Subsequently, the spores were washed with distilled water containing 0.05% Tween-80 and preserved in a 30% glycerin solution at −80 °C. Prior to utilization, the spore concentration was quantified using a hemocytometer and adjusted to 10^6^ colony-forming units per milliliter (CFU/mL).

### 2.2. The Expression, Purification, and Quantification of rPINB

Recombinant plasmids encoding rPINB were constructed following the protocol outlined [20] and subsequently transformed into *E. coli* BL21. Cultures containing the positive plasmids were expanded. Preliminary experiments identified the optimal IPTG concentration for induction as 10 μg/mL. The expression of rPINB was induced at 30 °C for 12 h. After induction, the cells were harvested by centrifugation, and the resulting cell pellet was resuspended in 10 mL of PBS. Cell lysis was achieved through sonication, and the target protein was purified using the method detailed in Tian et al. [20]. The purity of the rPINB was evaluated via SDS-PAGE, and its concentration was determined using the BCA assay.

### 2.3. The Inhibition of rPINB on A. flavus in Corn Meal Matrix

Non-mildewed corn was selected, ground, and allocated into five groups (n = 5 g/group), followed by high-temperature sterilization. Then, 5 mL of rPINB protein solution at concentrations of 0, 44.24, 88.48, 132.72, and 176.96 µg/mL was added to each group of corn flour, thoroughly mixed, and the surfaces were leveled (the rPINB concentration was designed based on the previous studies [19,20]). A spore solution of *A. flavus* (1 × 10^4^ spores per gram) was uniformly sprayed on the corn flour surfaces. Following a 6-day incubation period at 30 °C, the AFB1 content was determined as described by Tian et al. [17]. AFB1 was directly extracted from corn meal with 20 mL isoamyl acetate, and filtering samples through 0.22 µm syringe filters. After evaporation, the dried samples were resuspended in 1 mL methanol and determined by HPLC at a wavelength of 360 nm (Agilent, Santa Clara, CA, USA).

### 2.4. RNA-Seq and Real-Time Quantitative Polymerase Chain Reaction (RT-qPCR) Validation

*A. flavus* spores (1 × 10^6^ CFU/mL) were added to PDA medium with rPINB at 0, 44.24, 88.48, and 176.96 µg/mL and incubated at 30 °C for 48 h. Mycelia were then washed with PBS and frozen in liquid nitrogen. RNA was extracted using a total RNA extraction kit (Solarbio, Beijing, China), and sequenced by Shanghai Personal Biotech with an RNA-seq kit (Illumina, San Diego, CA, USA). Transcriptomic analysis was carried out using DESeq2, with significant gene expression differences set at *p* < 0.05 and |log2(FoldChange)| > 1. Enrichment analyses for GO terms (http://www.geneontology.org/) and KEGG pathways (http://www.genome.jp/kegg/, accessed 1 November 2024) were conducted on the differentially expressed genes.

RT-qPCR was utilized to validate RNA sequencing (RNA-seq) results by quantifying the expression levels of DEGs. The sequences of the gene primers were provided in Table S1. Total RNA was reverse transcribed into complementary DNA (cDNA) using the ReverTra Ace qPCR RT Master Mix with gDNA Remover (TOYOBO, Osaka, Japan). RT-qPCR analysis was conducted on the AB2000 system (Applied Biosystems, Foster City, California, USA) with a reaction volume of 20 µL, employing the SYBR^®^ Green Realtime PCR Master Mix (TOYOBO, Osaka, Japan). Relative gene expression levels were calculated using the 2^−ΔΔCt^ method [21].

### 2.5. The Construction of Mutant Strains

Homologous recombination was used to create deletion mutants of a target gene. The gene’s upstream and downstream regions were amplified with specific primers, while pyrg marker genes were amplified from the ANIp7 plasmid (refer to Table S2 for sequence details). These sequences were ligated and inserted into a pMD20 T-vector. Constructs were introduced into *A. flavus* NRRL 3357 protoplasts using CaCl_2_-PEG transformation. The protoplasts were then cultured on a hypertonic medium for three days to select gene knockout mutants (Figure S1). Confirmation of successful deletion was achieved by diagnostic PCR using primers orf/F and orf/R to amplify the target gene ORF.

### 2.6. Evaluation of the Response of the Mutant Strains to rPINB

The response of mutant strains to rPINB was evaluated by inoculating spores from wild-type (WT), *Δmfs1*, *Δmfs2*, and *Δmfs1Δmfs2* strains into PDB medium supplemented with rPINB protein at concentrations of 0 and 176.96 µg/mL, achieving a final spore density of 1 × 10^6^ CFU/mL. The cultures were maintained in conical flasks at 30 °C with shaking at 80 rpm. Samples of 100 µL were aseptically collected every two hours to monitor spore germination via the microscope. The spore germination was recorded when the germ tube extended beyond 50% of the spore’s diameter.

### 2.7. Analysis of Growth Characteristics in Mutant Strains

The 2 µL mutant spores from wild-type (WT), *Δmfs1*, *Δmfs2*, and *Δmfs1Δmfs2* strains were inoculated onto PDA medium and incubated at 30 °C. Colony diameters were measured daily. After 5 days, sections from the colony peripheries were excised to examine the growth morphology of conidial peduncles. Additionally, 5 mm × 5 mm samples from the colony edges were excised, immersed in 4% glutaraldehyde, and fixed for 24 h. *A. flavus* colonies were subsequently mounted on a platform using conductive tape, coated with gold, and analyzed via scanning electron microscopy (SEM) to observe the morphology of conidia and hyphae.

### 2.8. Sensitivity of Mutant Strains to Cell Membrane, Osmotic, and Oxidative Stress

To evaluate the sensitivity of mutant strains to stress, various agents were added to PDA medium: sodium dodecyl sulfate (SDS) for membrane stress (4%, 8%, 12%, and 16%), NaCl, KCl, and D-sorbitol for osmotic stress (0.4 M, 0.8 M, 1.2 M, and 1.6 M), and 4-nitroquinoline 1-oxide (4-NQO, 1, 2, 3, and 4 µg/mL) and hydrogen peroxide (H_2_O_2_, 2, 4, 6, and 8 mM) for oxidative stress. The PDA medium without any stress reagents functioned as the control group. After the medium solidified, spores from wild-type (WT), *Δmfs1*, *Δmfs2*, and *Δmfs1Δmfs2* strains were inoculated and incubated at 30 °C for three days, and the growth was observed and recorded

### 2.9. Evaluation of Aflatoxin B1 (AFB1) Production in Mutant Strains

*A. flavus* spores were inoculated into PDB medium at 10^5^ spores/mL and incubated at 30 °C for 10 days. Chloroform was added, incubated for 30 min, and allowed to stratify. The chloroform layer was extracted using a separatory funnel, evaporated, and AFB1 was dissolved in methanol, then filtered through a 0.22 µm membrane. AFB1 content was quantified via high-performance liquid chromatography (HPLC, Agilent, Santa Clara, CA) with post-column photochemical derivatization using an Agilent ZORBAX SB-C18 column (5 µm, 4.6 × 250 mm). The mobile phase was methanol and water (*v*/*v* = 60:40) at a 1 mL/min flow rate and 25 °C column temperature.

### 2.10. Statistics

All data are presented as the mean ± standard deviation (SD) of at least three independent biological replicates. Experimental data were expressed as the mean ± standard deviation from three independent experiments. Statistical differences between means were assessed using one-way ANOVA followed by Turkey’s test, with a significance threshold set at *p* < 0.05 among treatment groups. Data analysis and visualization were performed using GraphPad Prism 8.0 (GraphPad Software, San Diego, California, USA).

## 3. Results

### 3.1. The Inhibition of rPINB on A. flavus in Corn Meal Matrix

**Figure 1:**
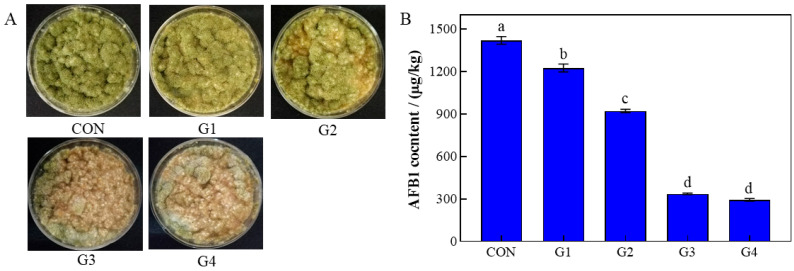
The inhibition of rPINB on the growth and AFB1 production of *A. flavus* on corn flour. (**A**): The growth of *A. flavus*; (**B**): The AFB1 production of *A. flavus*. G1, G2, G3, and G4 were the groups treated with 44.24, 88.48, 132.72, and 176.96 µg/mL rPINB, respectively. Different letters (a–d) indicate significant differences between groups (*p* < 0.05).

The impact of rPINB protein on the growth of *A. flavus* and AFB1 biosynthesis was assessed using a gradient concentration experimental system with sterile corn meal as the medium, as illustrated in Figure 1. The findings demonstrated that increasing concentrations of rPINB protein led to a reduction in *A. flavus* spore formation on the corn meal surface. Additionally, AFB1 production exhibited a concentration-dependent decrease, with a reduction of 79.3% observed in the G4 group. These results suggested that rPINB protein effectively inhibits fungal growth in corn meal, highlighting its potential application in agricultural product storage systems.

### 3.2. The Inhibition of rPINB Protein on the A. flavus Genome

#### 3.2.1. Differentially Expressed Genes (DEGs) in *A. flavus*

**Figure 2:**
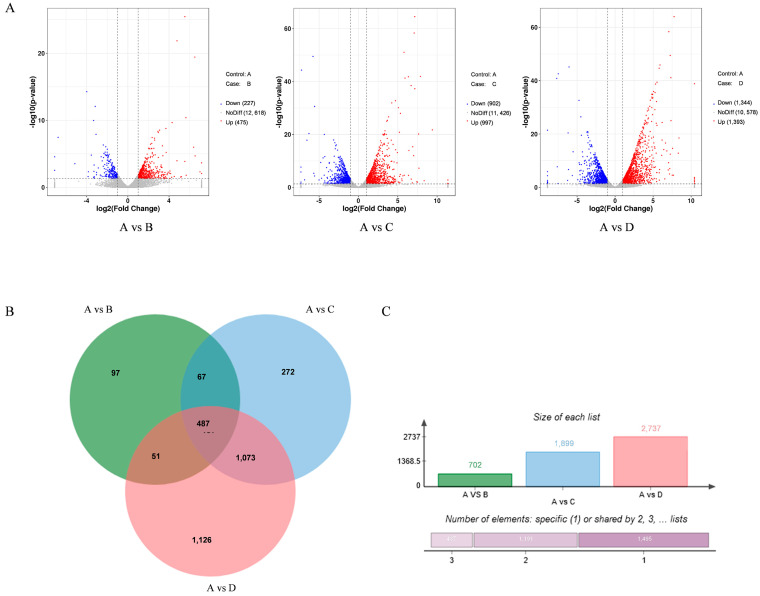
Venn diagram of differential genes. (**A**) The DEGs between control and rPINB treatment groups; (**B**) The common DEGs among all treatment groups; (**C**) The total DEGs of rPINB treatment groups. A: The control group; B, C, and D were the groups treated with 44.24 µg/mL, 88.48 µg/mL, and 176.96 µg/mL rPINB, respectively.

The DEG-Seq was used to analyze RNA-Seq data from *A. flavus*, with the results depicted in Figure 2. The number of DEGs in *A. flavus* treated with increasing concentrations of rPINB protein were 702, 1899, and 2737, respectively. Among these, 487 genes were co-expressed across all treatments. The unique gene expressions in the comparisons in A vs. B, A vs. C, and A vs. D were 97, 272, and 1,126, representing 13.82%, 14.63%, and 41.14% of the total DEGs. The analysis showed a positive correlation between the concentration of rPINB and the number of up-regulated and down-regulated genes.

#### 3.2.2. GO Enrichment Analysis in *A. flavus*

**Figure 3:**
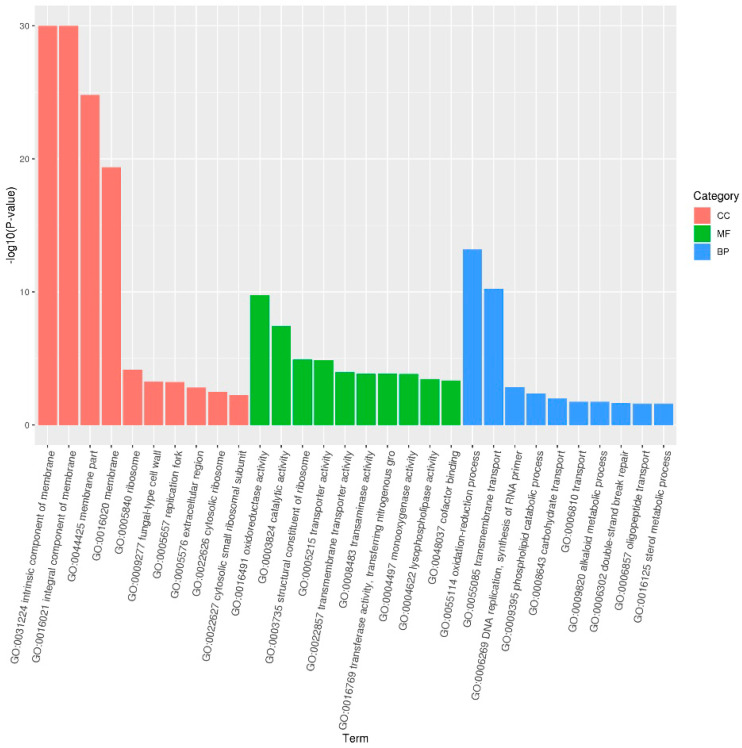
The GO differentially expressed gene enrichment analysis (A vs. D comparison). A: The control group; D: the groups treated with 176.96 µg/mL rPINB. *p*-value *<* 0.05. Terms with *p*-value < 0.05 are considered significantly enriched.

The GO analysis divides differential gene functions into Cellular Component (CC), Molecular Function (MF), and Biological Process (BP). In the A vs. D comparison, all differential genes were mapped to 2,981 subcategories. With a significance level of *p* < 0.05, 141 subcategories were significantly enriched: 26 in MF, 73 in CC, and 42 in BP. The top 10 significant GO terms for each category were illustrated in Figure 3. The DEGs were mainly enriched in cell membrane (GO:0031224, GO:0016021, GO:0044425, GO:0016020), redox processes (GO:0016491, GO:0055114), catalytic activities (GO:0003824), and transmembrane transport (GO:0055085). Results for A vs. B and A vs. C are shown in Figure S2.

The functional annotation of DEGs suggested that the rPINB protein may hinder *A. flavus* growth and toxin production by altering cell membrane integrity and permeability. From a cellular component perspective, significant enrichment was observed in cell membrane, cell wall, and ribosome-related components. Regarding biological functions, most genes were linked to redox processes, transmembrane transport, genetic information processing, and phospholipid degradation. From a molecular function standpoint, genes related to oxidoreductase activity, catalytic enzyme activity, ribosomal structure, and transmembrane transport were enriched. Overall, rPINB was shown to inhibit *A. flavus* growth and reduce AFB1 production by affecting the cellular membrane system, redox processes, and genetic information processing.

#### 3.2.3. Enrichment of KEGG Metabolic Pathway in *A. flavus*

**Figure 4:**
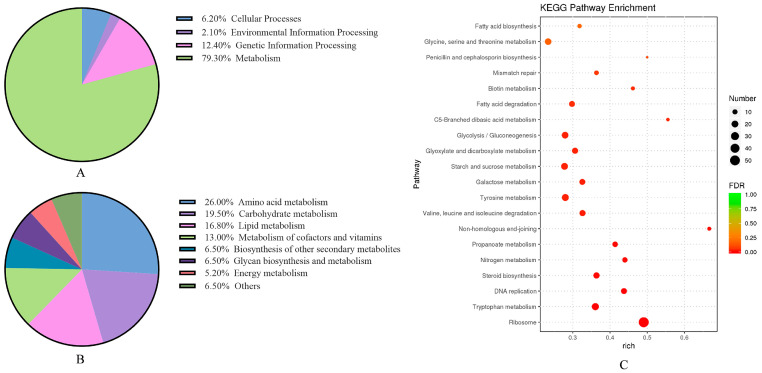
KEGG pathway enrichment analysis for *A. flavus* (A vs. D comparison). (**A**) Level 1 categories; (**B**) Level 2 subcategories; (**C**) top 20 enriched pathways. Dot size represents gene count; The FDR generally ranges between 0 and 1, with values closer to zero indicating more statistically significant enrichment.

In a cellular context, genes interact to perform biological functions. The KEGG database integrates gene function and genomic data to analyze gene relationships and regulation. DEGs between A vs. D were enriched in 98 metabolic pathways, mainly in metabolic and genetic information processing, comprising 91.8%. Amino acid, carbon source, and lipid metabolism made up 62.4% of these metabolic pathways. The top 20 enriched KEGG pathways were illustrated in Figure 4, including ribosome (afv03010), tryptophan metabolism (afv00380), and DNA replication (afv03030). KEGG analysis for A vs. B and A vs. C are detailed in Figure S3.

#### 3.2.4. Verification of Transcriptome Sequencing Results via RT-qPCR

**Figure 5:**
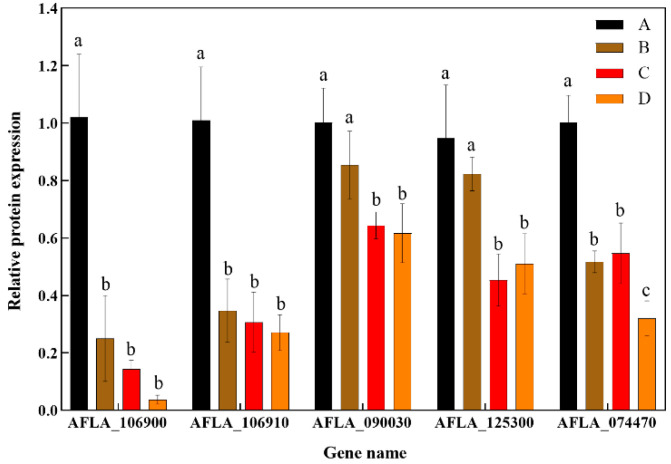
Relative expression levels of genes from transcriptome data. A: The control group; B: the group treated with 44.24 µg/mL rPINB; C: the group treated with 88.48 µg/mL rPINB; D: the group treated with 176.96 µg/mL rPINB. Different letters (a–c) indicate significant differences between groups (*p* < 0.05).

RT-qPCR was used to validate transcriptome sequencing results, as shown in Figure 5. Five DEGs from control and experimental groups were randomly selected, with primers designed for RT-qPCR analysis using *gpdA* as the reference gene. The results indicated that the expression patterns matched the sequencing data, confirming its accuracy and reliability of the sequencing data.

### 3.3. Impact of rPINB on the Transcriptional Expression of Genes Associated with Growth and Toxigenesis in A. flavus

An analysis of DEGs in *A. flavus*, influenced by the rPINB, revealed roles in cell wall synthesis, sporulation, transport activity, oxidative stress, and toxin synthesis. Table 1 demonstrated that transcriptional changes in *A. flavus* treated with rPINB were predominantly associated with cell membrane function. Notably, the genes AFLA_106900 and AFLA_106910, identified as *aflmfs1* and *aflmfs2*, respectively, may emerge as crucial genes through which rPINB inhibited the growth of *A. flavus*.

### 3.4. The Sensitivity of Δmfs1, Δmfs2 and Δmfs1Δmfs2 Mutants to rPINB

**Figure 6:**
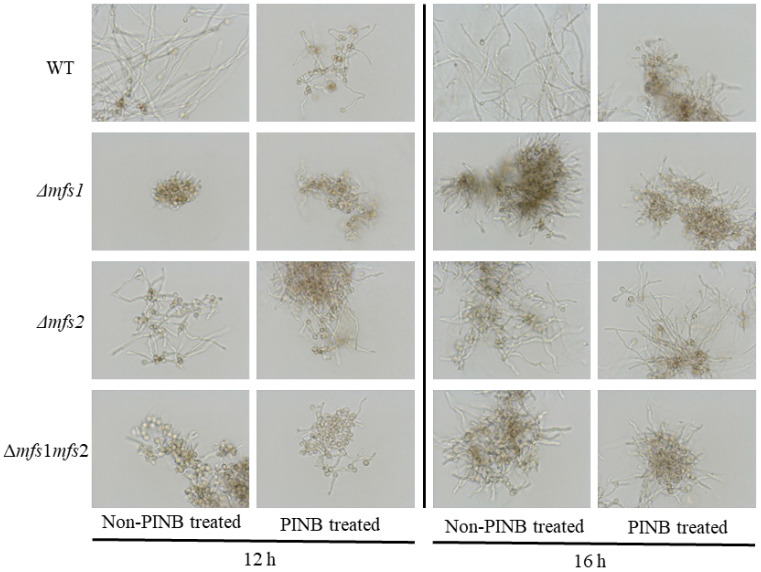
The effect of rPINB protein on the spore germination of *Δmfs1*, *Δmfs2,* and *Δmfs1Δmfs2* strains.

The growth of *A. flavus* mycelium treated with rPINB at concentrations of 0 and 176.96 µg/mL was observed at 12 h and 16 h, as illustrated in Figure 6. The rPINB significantly inhibited mycelial growth in the wild-type strain, but not significant inhibition in the *Δmfs1*, *Δmfs2*, and *Δmfs1Δmfs2* strains. These findings suggested that *mfs1* and *mfs2* may be crucial for the inhibition of *A. flavus* treated with rPINB.

### 3.5. Effects of mfs1 and mfs2 on the Growth of A. flavus

#### 3.5.1. The Growth of *Δmfs1*, *Δmfs2* and *Δmfs1Δmfs2* Mutant Strains on the PDA and YES

**Figure 7:**
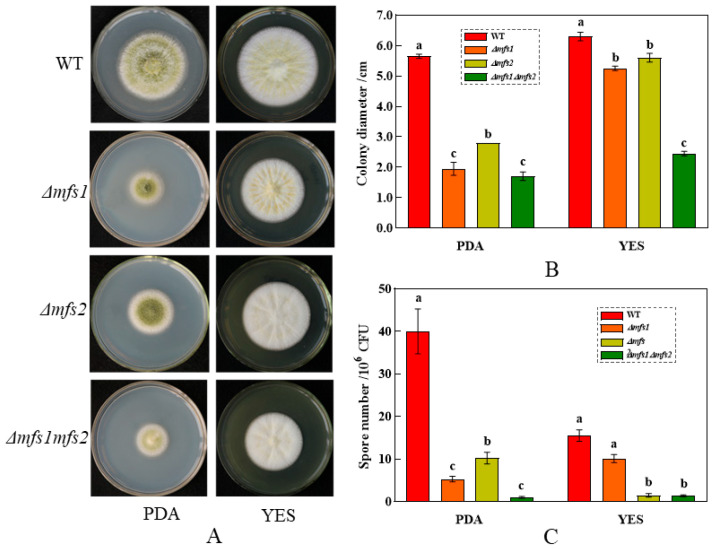
The *Δmfs1*, *Δmfs2*, and *Δmfs1Δmfs2* strains grown on the PDA and YES media. (**A**) Morphology of *A. flavus* colonies; (**B**) colony diameter; (**C**) spore production. a~c: the statistical annotations for groups in (**B**,**C**). Different letters (a–c) indicate significant differences between groups (*p* < 0.05).

The impact of *mfs1* and *mfs2* genes on *A. flavus* colonies is illustrated in Figure 7. On PDA medium, *Δmfs2* showed increased colony size (d*_Δmfs2_* = 28 mm) but lower spore production (10.23 × 10^6^ CFU/mL), whereas *Δmfs1* and *Δmfs1Δmfs2* had smaller colonies (d*_Δmfs1_* = 19.5 mm, d*_Δmfs1Δmfs2_* = 17 mm) and fewer spore production (1.00 × 10^6^ CFU/mL). On YES medium, *Δmfs2* exhibited a larger colony diameter (d*_Δmfs2_* = 56 mm) but minimal spore production (1.47 × 10^6^ CFU/mL), whereas *Δmfs1* had a smaller diameter (d*_Δmfs1_* = 52.5 mm) with spore production similar to WT strain (15.53 × 10^6^ CFU/mL). There was no significant spore production difference between *Δmfs2* and *Δmfs1Δmfs2* (*p* < 0.05). Thus, *mfs2* mainly influences spore production, while *mfs1* affects colony growth.

#### 3.5.2. The Microstructure of *Δmfs1*, *Δmfs2* and *Δmfs1Δmfs2* Mutant Strains

**Figure 8:**
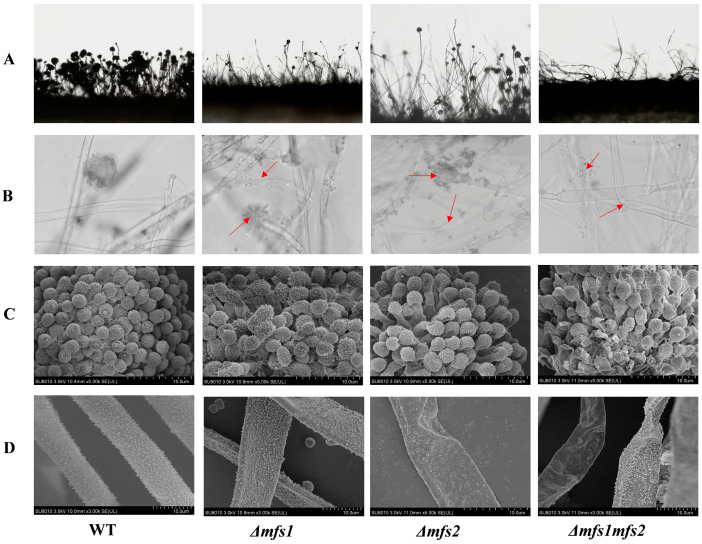
The microstructure of *Δmfs1*, *Δmfs2*, and *Δmfs1Δmfs2*. Optical microscopy observation of *A*. *flavus* conidiophores (**A**) and hyphae (**B**), scanning electron microscopy observation of *A. flavus* conidiophores (**C**), and hyphae (**D**). The bar of (**C**,**D**): d = 10 µm. (Red arrows mark the positions in the (**B**) that showed a significant difference from the control group).

The impact of *mfs1* and *mfs2* on the microscopic structure of *A. flavus* conidiophores and hyphae is shown in Figure 8. The WT strain has more conidiophores and heads compared to the *Δmfs1* and *Δmfs2* mutants, which show a significant reduction. The *Δmfs1Δmfs2* mutant almost entirely lacks distinct conidiophore heads, and the conidiophore surfaces in all mutants are wrinkled and uneven. SEM analysis revealed that the WT strain exhibited tightly packed, nearly spherical conidia, consistent hyphal thickness, and evenly distributed spiky protrusions. The *Δmfs1* mutant has loose conidiophore structures, some ellipsoidal conidia, and hyphal atrophy. The *Δmfs2* mutant also showed loose structures, conidial surface cavities, collapsed conidia, thin and wrinkled hyphae, and reduced, disordered protrusions, similar to the *Δmfs1Δmfs2* mutant.

### 3.6. Stress Assay

**Figure 9:**
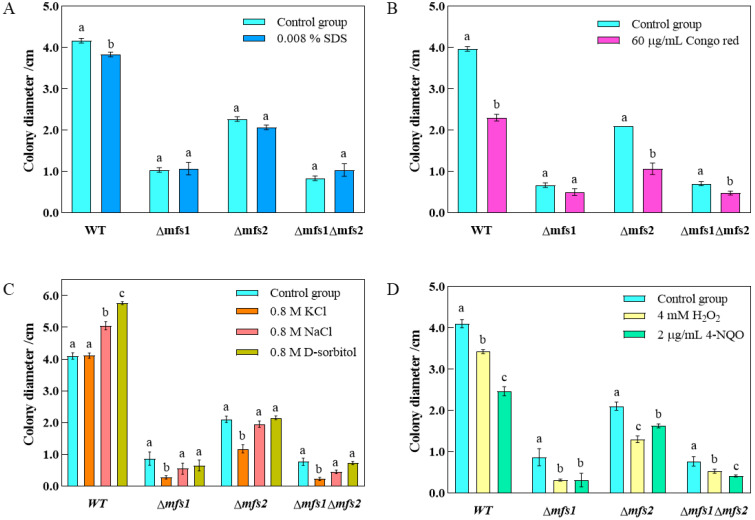
Sensitivity of *Δmfs1*, *Δmfs2*, and *Δmfs1Δmfs2* strains to the membrane stress (**A**), cell wall stress (**B**), osmotic pressure stress (**C**), and oxidative stress (**D**). Different letters (a–c) indicate significant differences between groups (*p* < 0.05).

The results of the sensitivity test for *Δmfs1*, *Δmfs2*, and *Δmfs1Δmfs2* mutants to stress agents were depicted in Figure 9. At SDS concentrations below 0.008%, the absence of *mfs1* and *mfs2* decreases sensitivity to membrane stress. For D-sorbitol concentrations under 0.8M, the deletion of *mfs1* and *mfs2* significantly reduces sensitivity to nonionic osmotic pressure. Similarly, at sodium chloride concentrations below 0.8 M, the absence of these genes delays the response to osmotic pressure stress. Additionally, the mutants exhibit reduced sensitivity to H_2_O_2_ and 4-NQO.

### 3.7. AFB1 Content

**Figure 10:**
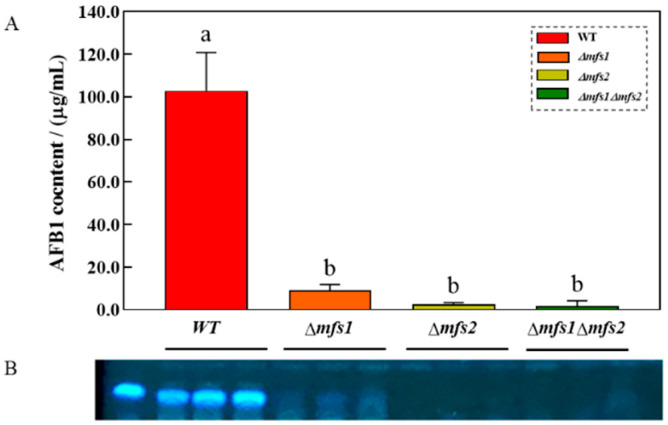
AFB1 content (**A**) and thin-layer chromatography (**B**) of *Δmfs1*, *Δmfs2*, and *Δmfs1Δmfs2* mutants. Different letters (a and b) indicate significant differences between groups (*p* < 0.05).

The production of AFB1 by WT, *Δmfs1*, *Δmfs2*, and *Δmfs1Δmfs2* mutants in PDB medium is depicted in Figure 10. After 6 d of static incubation at 30 °C, the AFB1 content in the *Δmfs1*, *Δmfs2*, and *Δmfs1Δmfs2* mutants was lowered more than 90% compared to the WT. This result suggested that the deletion of the *mfs1* and *mfs2* genes may indirectly affect AFB1 synthesis by disrupting cell membrane-related functions.

## 4. Discussion

Antifungal proteins, as emerging bioagents, have gained prominence as viable alternatives to chemical antifungal agents due to their environmentally friendly, safe, low-toxicity, and biodegradable characteristics. The PINB protein, recognized for its broad-spectrum antibacterial properties, exhibits potent inhibitory effects on both bacteria and fungi. In a previous study, we successfully developed a prokaryotic expression system for recombinant PINB (rPINB) and extracted rPINB with demonstrated antifungal activity [20]. In the current study, we observed that rPINB significantly reduced spore production of *A. flavus* in corn meal and decreased AFB1 production (Figure 1). Despite these findings, the molecular mechanism by which rPINB affects *A. flavus* remains to be elucidated.

Transcriptomics is a critical approach for elucidating the mechanisms of action of antifungal agents. To advance our understanding of the antifungal mechanism of rPINB against *A. flavus*, we conducted a transcriptomic analysis of *A. flavus* with and without rPINB treatment for the first time. The DEGs in *A. flavus* treated with rPINB were primarily associated with cell wall synthesis, spore formation, oxidative stress, toxin synthesis, and substance transport, suggesting that rPINB inhibits *A. flavus* through multiple pathways.

The cell wall acts as the first defense against external threats and is a key target for antibacterial drugs [22]. Glucan, chitin, and GPI proteins are the vital cell wall components. Down-regulation of genes related to α-1,3-glucan, chitin synthase, and GPI proteins may disrupt cell wall synthesis and function [23,24,25]. The decreased expression of the *Asg2* altered the morphology of mycelial cell wall and reduced spore production in *A. fumigatus* [26]. The invasiveness and toxicity of α-1,3-glucan to filamentous fungi had been confirmed in *Aspergillus fumigatus* [27]. *DCW1* is crucial for GPI-anchored protein synthesis in *Candida albicans*, and *chsD* is essential for chitin synthesis in *A. nidulans* [28,29,30]. Compared to the control group, DEGs such as *Ags2*, *Ags3*, *DCW1*, and *chsD* showed decreased expression with increasing rPINB concentration. Prior research indicated that rPINB-treated *A. flavus* hyphae appeared shriveled, and spores exhibited abnormal shapes [20]. Thus, reduced expression of *Ags2*, *Ags3*, *DCW1*, and *chsD* genes may hinder normal cell wall synthesis.

Spores are vital for fungal growth and environmental adaptation. The *vosA* gene is essential for spore development and maturation, as its absence results in spores lacking protective mechanisms. Studies have demonstrated that deletion of the *vosA* gene reduced the spores’ tolerance to heat and oxidative stress, while also compromising cell wall integrity [31,32,33]. Additionally, the hydrophobic DewA protein forms a protective layer on the spore surface, reducing water loss and aiding in desiccation resistance. The up-regulation of the hydrophobic protein gene *dewA* on the spore surface enhances the spores’ resistance to environmental stress [34,35]. Under stress induced by rPINB, the down-regulation of conidia-associated proteins and genes significantly impedes the formation of *A. flavus* spores. Conidia, which are a type of asexual spore in *A. flavus*, rely on these proteins and genes for proper development. Down-regulation disrupts spore development at multiple stages, potentially leading to incomplete spore wall formation or improper spore maturation.

As an aerobic organism, the mold utilizes oxygen within its cells to generate free radicals. These radicals exhibited potent oxidative capabilities, enabling them to target various biomacromolecules within fungal cells. For example, they could oxidize unsaturated fatty acids in cell membranes, initiating lipid peroxidation reactions that compromise membrane structure and function. This process altered membrane fluidity and increases permeability, resulting in the leakage of intracellular substances and disrupting normal cellular physiological functions. Additionally, radicals could modify proteins within cells, altering their structure and rendering them inactive, thereby affecting numerous enzymatic reactions. Furthermore, radicals had the potential to damage fungal DNA, leading to gene mutations and other issues that impact fungal growth, reproduction, and genetic stability [36,37]. Fungal cells possess various antioxidant substances and enzymes, such as superoxide dismutase (SOD), catalase (CAT), and glutathione peroxidase, to counteract these effects. SOD facilitates the conversion of superoxide anion radicals into hydrogen peroxide and oxygen. Subsequently, CAT decomposes hydrogen peroxide into water and oxygen, thereby diminishing free radical levels within the cell. This process is essential for maintaining cellular redox balance, safeguarding the cell from oxidative damage, and ensuring the normal survival and reproduction of fungi in aerobic environments. The presence of rPINB induces oxidative stress damage in the hyphae of *A. flavus* [20]. The gene expression levels associated with catalase activity were down-regulated, whereas those linked to superoxide dismutase exhibited slight up-regulation, aligning with prior research findings.

Aflatoxin biosynthesis is modulated by multiple pathways. For instance, the gene *aflD* encodes norsolorinic acid synthase, an enzyme crucial in the initial stages of aflatoxin biosynthesis that facilitates the conversion of polyketides to norsolorinic acid, a vital precursor in the synthesis of aflatoxins. The absence of *aflD* gene expression results in an early blockage of the aflatoxin biosynthetic pathway, leading to a marked reduction or complete cessation of aflatoxin production [38]. Shima’s research indicates that while *vrdA* is not classified as an aflatoxin biosynthesis gene, it nonetheless plays a role in the biosynthesis process within cells [39]. Jamali’s study further elucidates that the expression patterns of *aflO* and *aflQ* were strongly correlated with AFB1 production. Under specific growth conditions, the expression profiles of the *aflO* and *aflQ* genes within the aflatoxin biosynthesis pathway served as indicators of the AFB1-producing potential of *A. flavus* [40]. The down-regulation or absence of gene expressions such as *aflD*, *vrdA*, *aflQ*, and *aflO* in *A. flavus* could result in a marked reduction or complete cessation of AFB1 synthesis. The findings presented in Table 1 indicated that the expression levels of specific genes involved in aflatoxin biosynthesis were significantly down-regulated (*p*-value < 0.05), implying that the rPINB has the potential to mitigate aflatoxin production.

Previous research has identified the cell membrane as the primary target of the rPINB protein [41,42,43]. The cell membrane contains various transporters, including those from the Major Facilitator Superfamily (MFS) and ATP-Binding Cassette (ABC) transporters, which are instrumental in the uptake of extracellular nutrients and the expulsion of harmful metabolic byproducts [44,45]. These transporters are essential for maintaining normal cellular functions. In this study, most genes associated with MFS transporters, ABC transporters, exhibited down-regulation. Although it is well established that major facilitator superfamily (MFS) proteins were involved in transmembrane substance transport, their roles in pathogen growth and virulence remain underexplored. Empirical evidence suggested that the absence of *ChMfs1* resulted in hyphal distortion, swelling, and diminished spore production, while strains lacking *Pdmfs2* exhibited reduced spore production and a diminished capacity to infect citrus fruits [46,47,48]. Furthermore, the loss of cell membrane transporters *ctrA* and *ctrC* resulted in decreased superoxide dismutase and laccase activities, reduced cellular copper ion content, and impaired conidia formation and germination [49]. Additionally, siderophore transporters *Sit1* and *Sit2*, located within the plasma membrane, were considered potential virulence factors [50]. The *penT* gene boosts penicillin production by facilitating membrane transport [51]. Collectively, these findings underscore the critical role of MFS transporters in mold growth and the infection of plant hosts.

AFLA_106900 (*mfs1*) and AFLA_106910 (*mfs2*), annotated as MFS transporters in the Swiss-Prot database, were more responsive to rPINB protein compared to other transport-related genes. The *mfs1* showed significant down-regulation with fold changes of −7.11, -Inf, and -Inf in the comparisons of conditions A vs. B, A vs. C, and A vs. D; similarly, *mfs2* exhibited down-regulation with fold changes of −6.77, −7.31, and −7.61 across the same conditions. In this study, we generated *Δmfs1*, *Δmfs2*, and *Δmfs1Δmfs2* mutant strains for further investigation. The rPINB protein has been previously identified as an inhibitor of *A. flavus* mycelial growth. No significant differences in growth were observed among the *Δmfs1*, *Δmfs2*, and *Δmfs1Δmfs2* mutants treated with the rPINB protein after 16 h of culture. This finding suggests that the *mfs1* and *mfs2* genes are critical for mediating the effects of the rPINB.

The rPINB degraded *A. flavus* cell walls and membranes, leading to deformed and shriveled mycelia. The rPINB protein also elevated the reactive oxygen species and malondialdehyde in *A. flavus*, disrupting the redox system and reducing antioxidant enzyme activity, including catalase and superoxide dismutase, while altering GSSG and GSH levels [19,20]. In this study, *Δmfs1*, *Δmfs2*, and *Δmfs1Δmfs2* mutants had smaller colony diameters and lower spore production than the WT strain, with distorted and shrunken mycelia. SEM showed that mycelia of mutant strains were shrunken and folded, with looser conidial structures and deformed spores compared to WT. The mutants also showed less sensitivity to the membrane stress agents (SDS), osmotic stress agents (D-sorbitol, NaCl, and KCl), and oxidative stress agents (H_2_O_2_ and 4-NQO). Additionally, AFB1 production was significantly reduced. This study confirmed that the *mfs1* and *mfs2* genes were crucial targets through which the rPINB protein inhibits *A. flavus* growth and toxicity.

This study did not assess the practical application of rPINB protein in food preservation. The experiments will further explore its inhibitory effects on mold growth. Furthermore, analyzing the antibacterial mechanism of rPINB protein solely at the transcriptomic level lacks sufficient depth. Subsequent research will integrate metabolomics, proteomics, and other methodologies to comprehensively elucidate the antibacterial mechanism of rPINB protein against *A. flavus*. This approach aims to provide valuable insights for the application of rPINB protein as a plant-derived antifungal agent in food preservation.

## 5. Conclusions

The recombinant protein rPINB significantly inhibited the growth of *A. flavus* and the production of AFB1 in a dose-dependent manner on corn meal, achieving a reduction in aflatoxin production by 79.3%. Transcriptomic analysis revealed that rPINB disrupted gene expression associated with mycelial growth, impacting cell wall and membrane integrity, conidium formation, redox functions, and aflatoxin synthesis. Furthermore, the analysis identified *mfs1* and *mfs2* as critical for the inhibitory effects of rPINB on *A. flavus*. The mutant strains (*Δmfs1*, *Δmfs2*, *Δmfs1Δmfs2*) showed reduced sensitivity to rPINB, decreased growth, deformed conidia, shriveled mycelia, and lower sensitivity to the stress agents. AFB1 production was also significantly lower in these mutants. The discoveries presented novel ideas for the development of antifungal proteins and the control of aflatoxin contamination in agricultural products.

## Figures and Tables

**Table 1:** DEGs related to the growth and toxigenesis in *A. flavus*.

Gene ID	Gene Description/Homologous Gene	Log2FoldChange			Style
		A vs. B	A vs. C	A vs. D	
DEGs related to cell wall					
AFLA_116810	Alpha-1,3-glucan synthase/*Ags3*	−1.51 *	−2.35 *	−3.24 *	down
AFLA_134100	Alpha-1,3-glucan synthase/*Ags2*	−1.19	−1.33 *	−1.06 *	down
AFLA_095960	Chitin synthase D/*chsD*	−0.42	−1.11 *	−1.30 *	down
AFLA_002940	Endo-1,6-alpha-mannosidase/*DCW1*	−2.03 *	−1.61 *	−1.42 *	down
DEGs related to sporulation					
AFLA_074470	Spore development regulator/vosA	−0.98	−2.01 *	−3.11 *	down
AFLA_044790	Conidiation-specific family protein	0.38	−0.53	−1.19 *	down
AFLA_060780	Spore-wall fungal hydrophobin/dewA	1.43 *	1.33 *	1.28 *	up
DEGs related to redox function					
AFLA_100250	Catalase/*CAT*	−0.01	−0.66	−2.25 *	down
AFLA_056170	Catalase A/*catA*	−0.16	−0.91 *	−1.26 *	down
AFLA_075800	FAD-linked oxidoreductase/*patO*	−1.29 *	−1.14 *	−1.15 *	down
AFLA_033420	Superoxide dismutase/*sodB*	0.87 *	1.06 *	1.13 *	up
DEGs related to aflatoxin synthesis					
AFLA_046360	Acetyl-CoA carboxylase	−0.84	−0.77	−1.06 *	down
AFLA_125300	Short chain type dehydrogenase/*aflD*	−0.73	−2.92 *	−2.50 *	down
AFLA_090030	Aryl-alcohol dehydrogenase/*vrdA*	−1.25	−2.10 *	−1.87 *	down
AFLA_139200	Cytochrome P450 monooxygenase/*aflQ*	-Inf	-Inf	-Inf	down
DEGs related to transport function					
AFLA_106900	MFS antiporter/*qdr2*	−7.11 *	-Inf *	-Inf *	down
AFLA_106910	MFS membrane transporter	−6.77 *	−7.31 *	−7.61 *	down
AFLA_002590	MFS transporter/*mrr1*	−1.03 *	−2.57 *	−4.50 *	down
AFLA_116990	MFS monosaccharide transporter/*HXT3*	−1.28 *	−4.33 *	−4.43 *	down
AFLA_104430	ABC transporter/*tagD*	−2.13 *	−3.56 *	−3.31 *	down
AFLA_060080	ABC transporter/*FUM19*	−1.40 *	−2.90 *	−3.11 *	down

Note: A: The control group; B: the group treated with 44.24 µg/mL rPINB; C: the group treated with 88.48 µg/mL rPINB; D: the group treated with 176.96 µg/mL rPINB. * *p*-value < 0.05.

## Data Availability

The original contributions presented in this study are included in the article/Supplementary Materials. Further inquiries can be directed to the corresponding author.

## Supplementary Materials

The following supporting information can be downloaded at: https://www.mdpi.com/article/10.3390/foods14111903/s1, Table S1: Primer sequences required to RT-qPCR; Table S2: Primers used in the construction of the mutant strains; Figure S1: The mutants construction; Figure S2: GO enrichment; Figure S3: KEGG enrichment.
