# Supplementary material for: Transcriptome Sequencing Revealed an Inhibitory Mechanism of Recombinant Puroindoline B Protein on Aspergillus flavus
Source: Foods. 2025 May 27;14(11):1903. doi: 10.3390/foods14111903 (PMC12155302; doi:10.3390/foods14111903)
Supplement: Supplementary file 1 [file foods-14-01903-s001.zip › Table S1.docx]

| Table S1 Primer sequences required to RT-qPCR | |
| --- | --- |
| Gene name | Primers |
| AFLA_106910 | F: CACAATCGGCTACCTAATGG |
|  | R: AGCAAAGGGCACAGAACC |
| AFLA_106900 | F: TACAGGAGCAGGTGTCATCG |
|  | R: CAGGGTCTCAGGAAGTAATAGGA |
| AFLA_090030 | F: TAACAGGGAGGTGGTTGATT |
|  | R: CGATTTCTTCGGATTTGC |
| AFLA_125300 | F: CCAGCAGTCAACCCAAGT |
|  | R: TCAACAATCAACCACCTCC |
| AFLA_074470 | F: CAGGTGGTAGAGCGAGGAA |
|  | R: TGATGGACGCCAAATGAG |
