# Supplementary material for: Transcriptome Sequencing Revealed an Inhibitory Mechanism of Recombinant Puroindoline B Protein on Aspergillus flavus
Source: Foods. 2025 May 27;14(11):1903. doi: 10.3390/foods14111903 (PMC12155302; doi:10.3390/foods14111903)
Supplement: Supplementary file 1 [file foods-14-01903-s001.zip › Table S1.pdf]

Table S1 Primer sequences required to RT-qPCR

Gene name	Primers
AFLA_106910	F: CACAATCGGCTACCTAATGG
	R: AGCAAAGGGCACAGAACC
AFLA_106900	F: TACAGGAGCAGGTGTCATCG
	R: CAGGGTCTCAGGAAGTAATAGGA
AFLA_090030	F: TAACAGGGAGGTGGTTGATT
	R: CGATTTCTTCGGATTTGC
AFLA_125300	F: CCAGCAGTCAACCCAAGT
	R: TCAACAATCAACCACCTCC
AFLA_074470	F: CAGGTGGTAGAGCGAGGAA
	R: TGATGGACGCCAAATGAG
