# Supplementary material for: Transcriptome Sequencing Revealed an Inhibitory Mechanism of Recombinant Puroindoline B Protein on Aspergillus flavus
Source: Foods. 2025 May 27;14(11):1903. doi: 10.3390/foods14111903 (PMC12155302; doi:10.3390/foods14111903)
Supplement: Supplementary file 1 [file foods-14-01903-s001.zip › Table S2.pdf]

Table S2 Primers used in the construction of the mutant strains

Gene	The primer
mfs1-1F	CCGTTAGGCTTCATCCC
mfs1-1R	CGCGTTCTCGAGGAAGTTGCGCCTGGTCCGTGTTCGA
mfs1-2F	GTGTAACGGTATTGACTAAAAGGGGCCGAGGCTGACTTAGACG
mfs1-2R	CAAACACTGGGAGAAGATGGA
mfs1-3F	CACAATCGGCTACCTAATGG
mfs1-3R	CAGAGCAAAGGGCACAGAA
mfs1-4F	ACCGAGCCTTCTTTCTGTTGG
mfs1-4R	ACCGCTTGGTTTATCCTGTGAG
mfs2-1F	GGCTGTGGGTGTCGGAATA
mfs2-1R	CGCGTTCTCGAGGAAGTTGCGCAAAGGGAATCAAGGTGC
mfs2-2F	GTGTAACGGTATTGACTAAAAGGGTCGAACACGGACCAGGC
mfs2-2R	CGGAAGAACAGGGAATGAGT
mfs2-3F	TTTTCTCACCGTTGTCTTCCA
mfs2-3R	ATCCCTGACTCGGCACCCT
mfs2-4F	CAGACAGCCACTCCGTTTCA
mfs2-4R	GCCGCTTATTTCACTTTCAGG
pyrg-F	GCAACTTCCTCGAGAACGCG
pyrg-R	CCCTTTTAGTCAATACCGTTACAC
