# Supplementary figures and images for: Transcriptome Sequencing Revealed an Inhibitory Mechanism of Recombinant Puroindoline B Protein on Aspergillus flavus
Source: Foods. 2025 May 27;14(11):1903. doi: 10.3390/foods14111903 (PMC12155302; doi:10.3390/foods14111903)

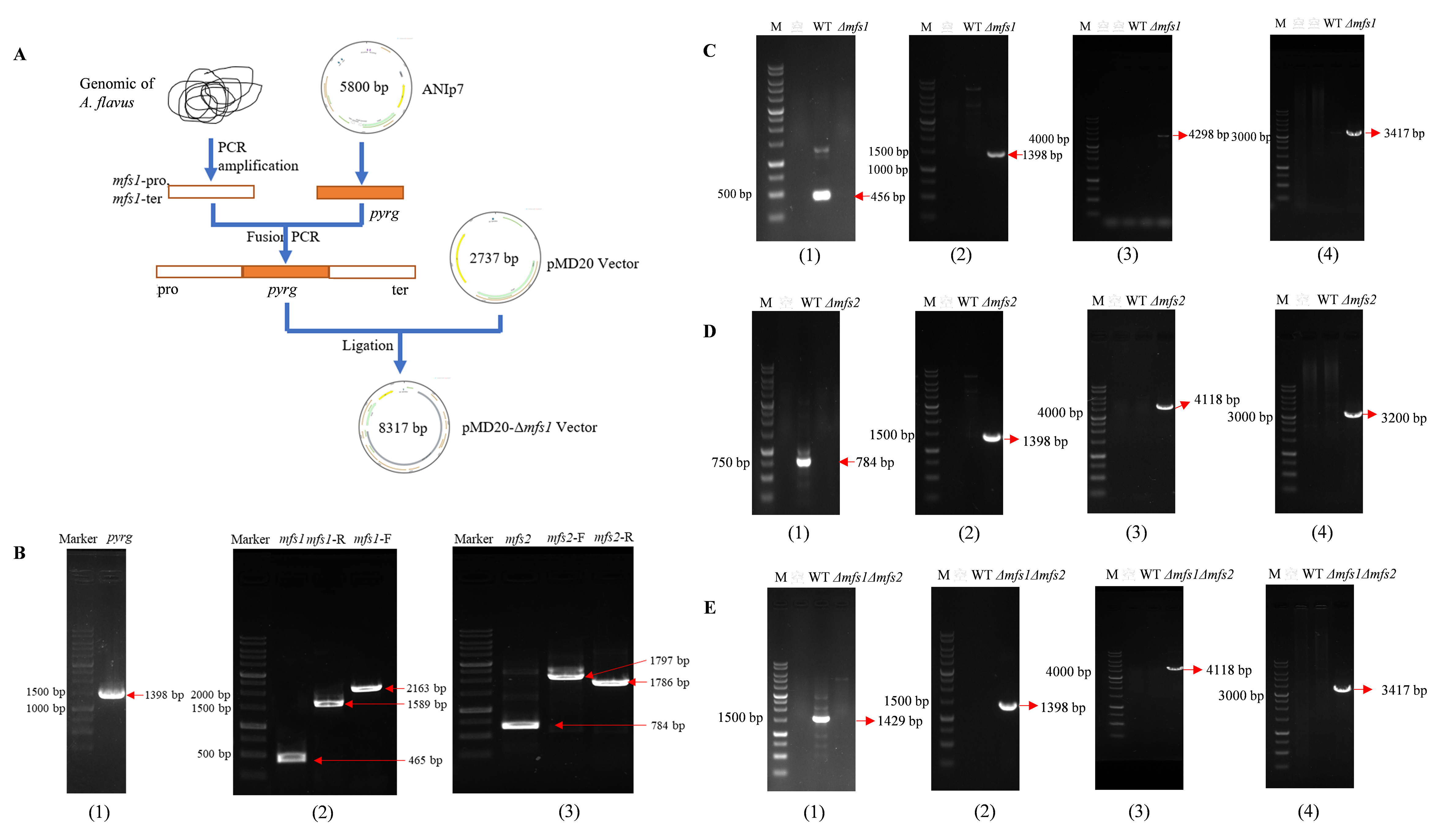

Supplement: Supplementary file 1 [file foods-14-01903-s001.zip › Figure S1 The mutants construction.png]

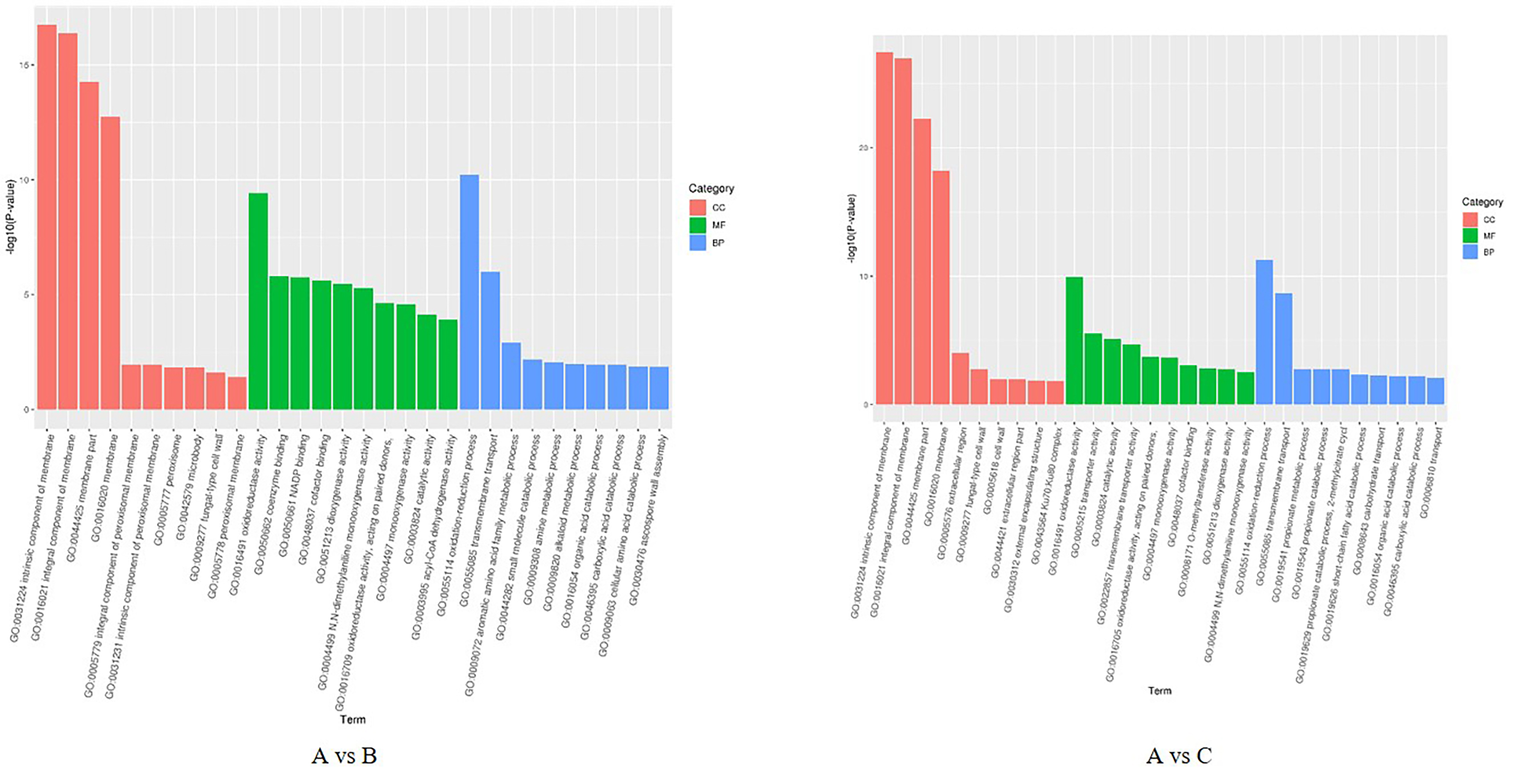

Supplement: Supplementary file 1 [file foods-14-01903-s001.zip › Figure S2 GO enrichment.jpg]

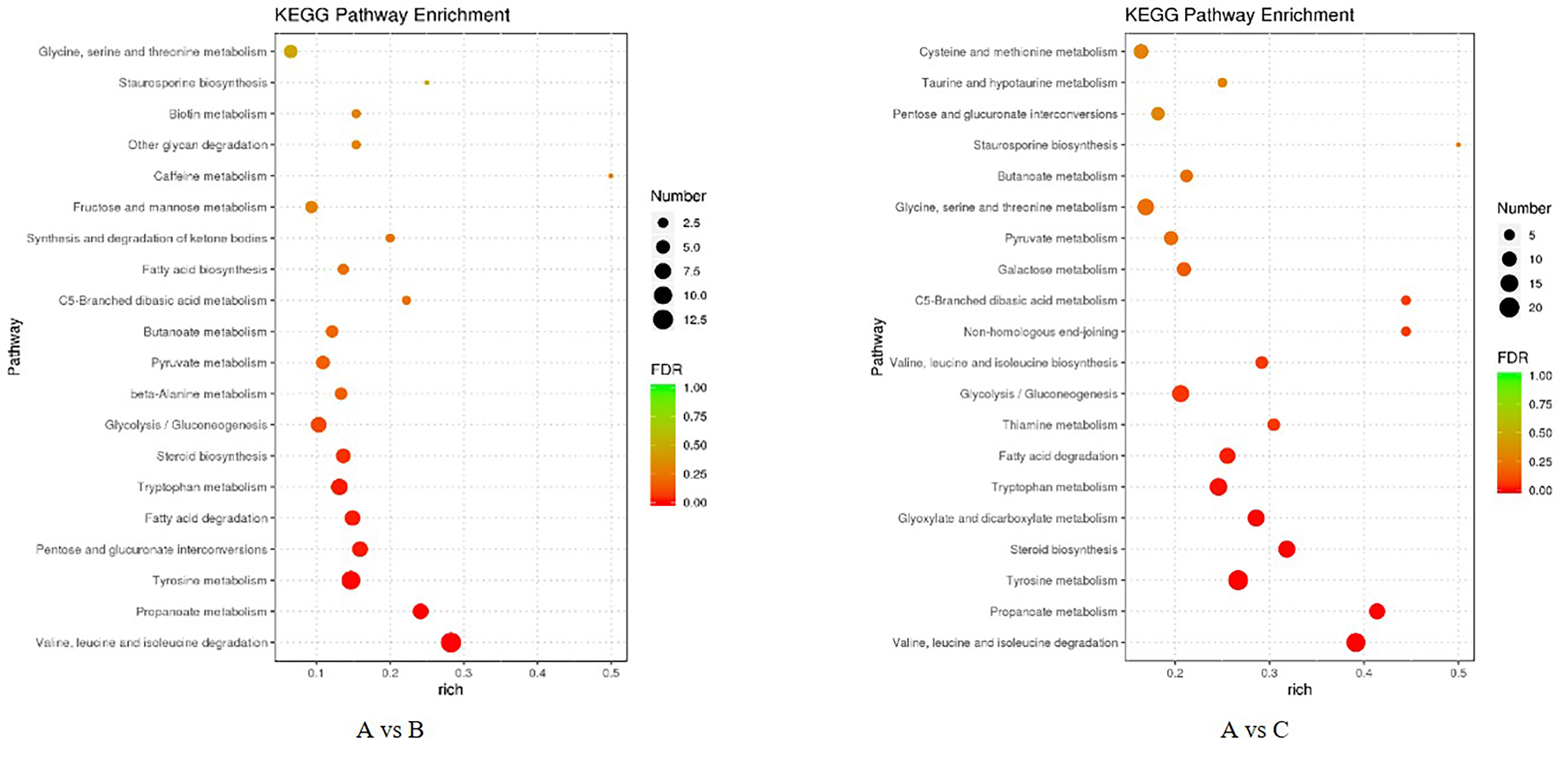

Supplement: Supplementary file 1 [file foods-14-01903-s001.zip › Figure S3 KEGG enrichment.jpg]
